# TNFα-TNFR2 signaling pathway in control of the neural stem/progenitor cell immunosuppressive effect: Different experimental approaches to assess this hypothetical mechanism behind their immunological function

**DOI:** 10.1186/s13287-020-01816-2

**Published:** 2020-07-22

**Authors:** Sara Shamdani, Georges Uzan, Sina Naserian

**Affiliations:** 1 INSERM UMR-S-MD 1197, Hôpital Paul Brousse, Villejuif, France; 2 CellMedEx, Saint Maur Des Fossés, France; 3 Paris-Saclay University, Villejuif, France

**Keywords:** TNF-TNFR2 signaling pathway, Immune checkpoint, Immunosuppression, Immunoregulation, Neural progenitor cells, Neural stem cells

## Abstract

**Background:**

Stem cells have a vast range of functions from tissue regeneration to immunoregulation. They have the ability to modulate immune responses and change the progression of different inflammatory and autoimmune disorders. Tumor cells share many characteristics of stem/progenitor cells too. Both can inhibit effector T cells and other immune cells, while inducing regulatory T cells (T regs), thus, reducing the production of pro-inflammatory cytokines and increasing the production of anti-inflammatory ones. In this context, some cytokines like TNFα are able to control the direction of the immune response. TNF-TNFR signaling plays a dual role: while the interaction of TNFα with TNFR1 mediates pro-inflammatory effects and cell death, its interaction with TNFR2 mediates anti-inflammatory effects and cell survival.

**Main body:**

We think the expression of TNFR2 confers a level of immunomodulatory properties to its expressing cell and this could be crucially important, particularly, for stem/progenitor and tumor cells. This idea has been already proven in many TNFR2^+^ cells. Different immunosuppressive cells like T regs, regulatory B cells (B regs), myeloid-derived suppressor cells (MDSCs), mesenchymal stem cells (MSCs) and endothelial progenitor cells (EPCs) express TNFR2 and are able to suppress immune cells in presence of TNFα. The other category of rare cells that express TNFR2 is neural cells (NCs). Although little is known about the immunological function of these latter cells, few studies showed their progenitors are able to suppress T cells. Therefore, we hypothesize that the immunosuppressive effect of neural stem cells (NSCs) is potentially TNFα-TNFR2 dependent.

**Conclusions:**

NSCs are among the rare cells that express TNFR2 marker and are able to supress T cells. We believe TNFα-TNFR2 immune checkpoint signaling pathway could be responsible for this immunosuppressive effect.

## Background

Tumor necrosis factor alpha (TNFα) is a pro-inflammatory cytokine that could modulate both pro- and anti-inflammatory properties [1]. TNFα interacts with two distinct transmembrane receptors, TNFR1 (CD120a) and TNFR2 (CD120b). TNFR1 is expressed ubiquitously on almost all cells, and its binding to TNFα will lead to apoptosis and eventually cell death. TNFR2, however, is limitedly expressed on certain cells including immune cells, endothelial cells (ECs), MSCs and neural cells (NCs), and its interaction with TNFα leads to proliferation and cell survival [2]. Is has been evidenced that TNFα increases the expression of several pro-angiogenic factors such as vascular endothelial growth factor (VEGF), basic fibroblast growth factor (bFGF), and IL-8 in ECs [3]. Moreover, it has been shown that TNFα is involved in proliferation, neuronal differentiation, and neurogenesis [4]. Indeed, TNFα-TNFR2 axis generally supports the protective mechanisms, and inversely, TNFα-TNFR1 axis is involved in deleterious mechanisms. For example, it has been shown that TNFR1 mediates myocardiac ischemic injuries and has toxic effects in models of myocardial infarction; however, TNFR2 signaling is protective in infract myocardium, heart ischemic injuries, and aging [5]. Moreover, unlike interaction through TNFR1, TNFα-TNFR2 signaling can increase the anti-inflammatory and immunosuppressive mechanisms [6].

## Main text

Observing particular immune and physiological reactions of cells expressing TNFR2 molecule encouraged us to study its role in limited cells that express this marker and interestingly found out that in almost all TNFR2^+^ cells, there is at least one kind of immunoregulatory function. We and others have clearly shown that many immunosuppressive cells like T regs, B reg, and MDSCs express TNFR2, and this is directly related to the efficiency of immunosuppression mostly via modulating the secretion of anti-inflammatory cytokines [6–8].

Furthermore, we have been investigating on ECs, particularly, endothelial progenitor cells (EPCs) that are among the rare cells expressing TNFR2 and which TNFα is critically important for their activation, migration, and angiogenic activities. We recently demonstrated that cord blood EPCs (CB-EPCs) and adult peripheral blood EPCs (APB-EPCs) have immunosuppressive and immunomodulatory functions against T cells. Additionally, we showed that CB-EPCs are able to induce new functional vessels in xenogeneic ischemic mice and are tolerated and resistant in several tissues after their first administration. Very interestingly, our data proved that the EPC immunosuppressive effect was entirely TNFα-TNFR2 dependent [9, 10].

Likewise, we have shown in several in vitro and in vivo studies that MSCs strongly suppress immune cells in innate and adaptive immune systems. MSCs inhibit effector T cell (T eff) proliferation and function in all autologous, allogenic, and xenogeneic conditions while promoting and inducing T regs (iT regs). In consequence, they decrease the secretion of pro-inflammatory cytokine [11]. MSC immunoregulatory function can be changed when exposed to an inflammatory microenvironment. TNFα and other inflammatory cytokines have been shown to actively prime MSCs towards more immunosuppressive phenotypes [12]. We have recently investigated the involvement of the TNFα-TNFR2 signaling pathway in the immunosuppressive effect of MSCs. Our results demonstrated for the first time that the TNFα-TNFR2 signaling pathway plays a critical role in MSC immunomodulatory effect. Unlike WT-MSCs, TNFR2 KO-MSCs were remarkably less able to suppress T cells, down-modulate T cell activation markers and pro-inflammatory cytokine secretion, and induce active T regs [13].

The other rare cells that express TNFR2 is neural cells (NCs). It has been shown that TNFα signaling has also both positive and negative effects on neurogenesis and is required to moderate the negative impact of cranial irradiation on hippocampal neurogenesis and neuroinflammation [14]. Very interestingly, it has been reported that neural stem/progenitor cells (NSCs) from mouse, rat, and pig are demonstrating immunosuppressive effect against anti-CD3/CD28 polyclonal activated effector T cells [15].

Based on solid results that correlate the expression of TNFR2 and immunoregulatory function, we hypothesize that the immunosuppressive function of NSCs could be also TNFα-TNFR2 dependent. Thus, we think it is necessary to answer this question by neuroscience experts. In order to do that, we suggest co-culturing different NCs including NSCs with different immune cells including T cells (CD4^+^ and CD8^+^ populations) and eventually macrophages and NK cells and to observe if the immunosuppressive effect observed by NSCs is partially or entirely TNFR2 dependent.

**Fig. 1:**
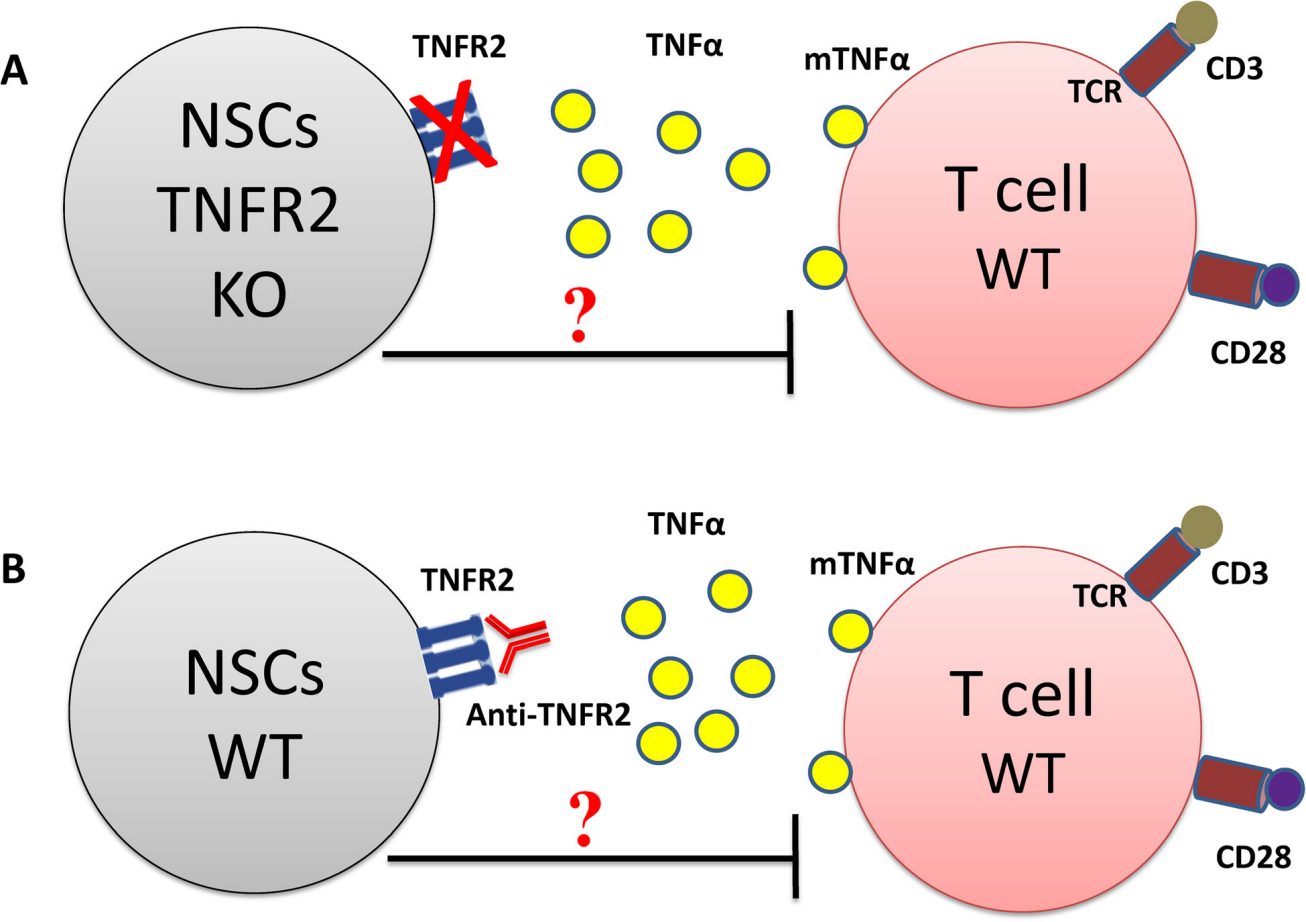
Interfering with TNFα-TNFR2 signaling pathway via blocking the receptor. This schematic depicts our primary hypothesis based on the direct involvement of TNFα-TNFR2 axis in immunomodulatory functions observed by NSCs. In order to validate this hypothesis, we should hamper this signaling pathway either, **a**) by knocking out the TNFR2 gene or harvesting NSCs from TNFR2 KO mice or **b**) by neutralizing TNFR2 expressed on NSCs using anti-TNFR2 monoclonal anti-body. Then, after creating these two co-culture conditions, one should assess if NSCs are still able to exert their immunosuppressive function on T cells. TCR, T cell receptor; mTNFα, membrane form of TNFα

**Fig. 2:**
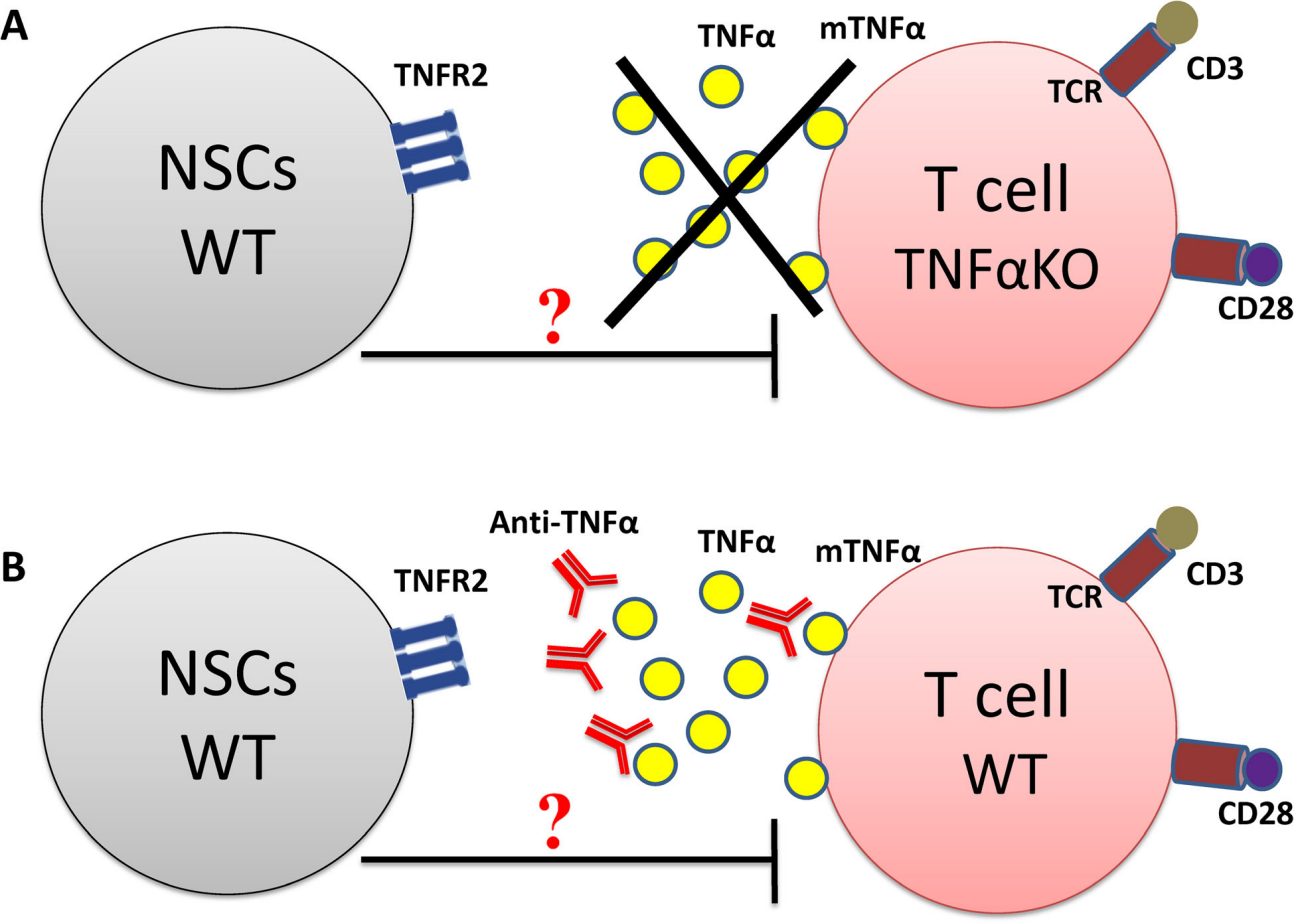
Interfering with TNFα-TNFR2 signaling pathway via blocking the ligand. This schematic depicts our primary hypothesis based on the direct involvement of TNFα-TNFR2 axis in immunomodulatory functions observed by NSCs. In order to validate this hypothesis, we should hamper this signaling pathway firstly, **a**) by using T cells harvested from TNFα KO mice that are incapable of TNFα production. In this setting TNFR2 (receptor) is expressed by NSCs but no TNFα (ligand) will be produced by T cells. **b**) Secondly, by using anti-TNFα monoclonal anti-body to neutralize the membrane and secreted form of TNFα. Then, after creating these two co-culture conditions, one should assess if NSCs are still able to exert their immunosuppressive function on T cells. TCR, T cell receptor; mTNFα, membrane form of TNFα

In order to prove that, the TNFα-TNFR2 signaling pathway must be interfered either by knocking out TNFR2 gene in neural cells or using NSCs harvested from TNFR2 KO mice (Fig. 1a) or using anti-TNFR2 monoclonal anti-body (anti-TNFR2-mAb) to block the receptor (Fig. 1b). This approach should be reinforced by using T cells derived from TNFα KO mice (Fig. 2a) or application of anti-TNFα mAb to block the ligand (Fig. 2b). In addition, it will be very important to assess if the immunosuppressive effect demonstrated by NSCs is correlated to their stemness features and the passage/age (population doubling level) of the cells or even mature NCs are able to suppress immune cells through TNFα-TNFR2 axis.

In some cancers, the number of neurons is increased, which suggests that a potential process of neurogenesis occurs and this phenomenon could support tumor development and progression. Answering these questions is crucial since administration of anti-TNFR2 agents (TNFR2 antagonist) seems to be an effective way in cancer immunotherapy. Therefore, it is very important to understand which cells are directly affected by this kind of treatment and what will be the consequences in every type of TNFR2 expressing cells.

## Conclusions

Through different in vitro and in vivo experimental approaches, TNFα-TNFR2 immune checkpoint signaling pathway was reported important for controlling the immunoregulatroy functions of almost all TNFR2^+^ cells including stem/progenitor cells. This axis can modulate different immunological aspects of stem cells such as the production of different anti-inflammatory cytokines. We strongly believe that targeting TNFR2 using its proper antagonist is an effective way to treat cancer as it efficiently controls immunosuppression, tumor angiogenesis, tumor neurogenesis, and survival. Therefore, it is critical to understand what will be the exact effect of anti-TNFR2 treatment in every cell expressing this immunoregulatory marker. NSCs are one of those TNFR2 expressing cells that have not been sufficiently studied for their immunomodulatory features. We hypothesize that there might be a direct relationship between the expression of TNFR2 and the immunosuppressive effect of NSCs.

## Data Availability

Not applicable

## Abbreviations

APB: Adult peripheral blood
B reg: Regulatory B cell
bFGF: Basic fibroblast growth factor
CB: Cord blood
CD: Cluster of differentiation
ECs: Endothelial cells
EPCs: Endothelial progenitor cells
IL-8: Interleukin-8
mAb: Monoclonal anti body
MDCSs: Myeloid-derived suppressive cells
MSCs: Mesenchymal stem cells
NCs: Neural cells
NSCs: Neural stem cells
T eff: Effector T cell
T reg: Regulatory T cell
TNFR1: Tumor necrosis factor receptor 1
TNFR2 KO: Tumor necrosis factor receptor 2 knock-out
TNFR2: Tumor necrosis factor receptor 2
TNFα: Tumor necrosis factor alpha
VEGF: Vascular endothelial growth factor
